# Phospholipid arrays on porous polymer coatings generated by micro-contact spotting

**DOI:** 10.3762/bjnano.8.75

**Published:** 2017-03-27

**Authors:** Sylwia Sekula-Neuner, Monica de Freitas, Lea-Marie Tröster, Tobias Jochum, Pavel A Levkin, Michael Hirtz, Harald Fuchs

**Affiliations:** 1 Institute of Nanotechnology (INT) and Karlsruhe Nano Micro Facility (KNMF) Karlsruhe Institute of Technology (KIT), 76344 Eggenstein-Leopoldshafen, Germany; 2 Institute for Photon Science and Synchrotron Radiation (IPS), Laboratory for Applications of Synchrotron Radiation (LAS), Karlsruhe Institute of Technology (KIT), 76344 Eggenstein-Leopoldshafen, Germany; 3 Institute of Toxicology and Genetics (ITG), Karlsruhe Institute of Technology (KIT), 76344 Eggenstein-Leopoldshafen, Germany; 4 Institute of Organic Chemistry, Karlsruhe Institute of Technology, 76021 Karlsruhe, Germany; 5 Physikalisches Institut and Center for Nanotechnology (CeNTech), Universität Münster, 48149 Münster, Germany

**Keywords:** microcontact cantilever spotting, phospholipids, polymeric porous support, polymethacrylate

## Abstract

Nanoporous poly(2-hydroxyethyl methacrylate-*co*-ethylene dimethacrylate) (HEMA-EDMA) is used as a 3D mesh for spotting lipid arrays. Its porous structure is an ideal matrix for lipid ink to infiltrate, resulting in higher fluorescent signal intensity as compared to similar arrays on strictly 2D substrates like glass. The embedded lipid arrays show high stability against washing steps, while still being accessible for protein and antibody binding. To characterize binding to polymer-embedded lipids we have applied Streptavidin as well as biologically important biotinylated androgen receptor binding onto 1,2-dipalmitoyl-*sn*-glycero-3-phosphoethanolamine-*N*-(cap biotinyl) (Biotinyl Cap PE) and anti-DNP IgE recognition of 2,4-dinitrophenyl[1,2-dipalmitoyl-*sn*-glycero-3-phosphoethanolamine-*N*-[6-[(2,4-dinitrophenyl)amino]hexanoyl] (DNP)] antigen. This approach adds lipid arrays to the range of HEMA polymer applications and makes this solid substrate a very attractive platform for a variety of bio-applications.

## Introduction

Starting with the creation of high density peptide microarrays in the 1990s [1] the development of arrays with DNA molecules and antibodies has produced a range of platforms for functional determination studies of molecules [2–4]. Nowadays, commercially available micro- and nanoarrays in a variety of design are readily available. Lipid arrays, however, are a comparably novel platform and still not as well-developed. Studies using lipid micro-/nanoarrays are often hampered due to the lack of widely available technologies for lipid deposition [5] and the detection of lipid–protein interactions [6]. Increased biological importance of lipid arrays, demonstrated by the growing number of studies on autoimmune diseases such as multiple sclerosis [7–8], emphasizes the need for sustained progress in this field.

The work presented here demonstrates the potential of nanoporous poly(2-hydroxyethyl methacrylate-*co*-ethylene dimethacrylate) (HEMA-EDMA) as a novel advantageous substrate for lipid arrays. Porous HEMA support, with pore size distribution in the range of 150 nm , has already demonstrated advantages in pattern definition, spot homogeneity, and consistent spot dimensions for different dye sensors (phloxine B and bromophenol blue) spotted in ethanol based inks [9]. In the present approach, the pores of the HEMA-EDMA support act as a mesh that contains lipid ink in a confined space, providing high pattern definition, and presenting more binding sites than would be available on a flat substrate [10]. The main phospholipid component in our ink mixture is 1,2-dioleoyl-*sn*-glycero-3-phosphocholine (DOPC, *T*_m_ = −16.5 °C) which allowed for the control of ink fluidity under humidity controlled conditions [11–12]. Using quill-like pens (SPTs, short for surface patterning tool [13]) for lipid ink deposition permitted the reduction of the volume of phospholipids needed for array generation, as compared to other spotting techniques like ink jet printing, without decrease in the quality of the array or reduction of binding sites for analyte molecules. On the contrary, higher resolutions and smaller feature sizes are obtained with the SPTs in comparison with common ink jet procedures. We previously demonstrated the use of SPTs for the deposition of water-based click-chemistry inks [10,14–15] where CuAAC mediated covalent immobilization of ink molecules and embedment’s within the polymer mesh makes the pattern highly stable in solution [10]. To determine if lipids deposited on nanoporous HEMA-EDMA polymer could be used to generate functional arrays we selected lipid–protein pairs applied in previous settings: Biotin-Cap-PE and streptavidin labeled with Cy3 dye (STV-Cy3) as a simple protein model; and DNP-cap-PE with anti-DNP IgE as a model for allergen/antibody recognition. These interactions are well-characterized for biomimetic lipid membranes on flat supports [16–17] and there, interactions occur without any special requirements, like pretreatment with co-activating molecules. For a more complex protein, the lipid/HEMA-EDMA substrate system was also characterized for the binding properties of biotinylated androgen receptor (AR^biot^) [18] onto Biotin-cap-PE arrays in the presence of a hormone. We have chosen AR as a test protein because of its biological importance: the transcriptional activity of this steroid receptor is crucial not only for the normal development of the prostate in males, but it is also vital in the progression of prostate cancer [19]. AR contributes to breast cancer progression and development [20] and its mutation is involved in the progression of neurodegenerative diseases, such as the X-linked spinal and bulbar muscular atrophy [21]. In order to better understand and characterize the behavior and mechanism of action of this receptor new approaches are needed to facilitate studies with AR. Embedding AR in a lipid layer within HEMA-EDMA mesh might facilitate studies relying not only on fluorescence microscopy techniques but also on spectroscopy, to evaluate coactivators, co-chaperones [22] and even novel antiandrogens affinity to the receptor [6] and effects on the receptor’s poly Q-extended mutations [23].

## Results and Discussion

### Microchannel cantilever spotting (µCS) with phospholipids

**Figure 1:**
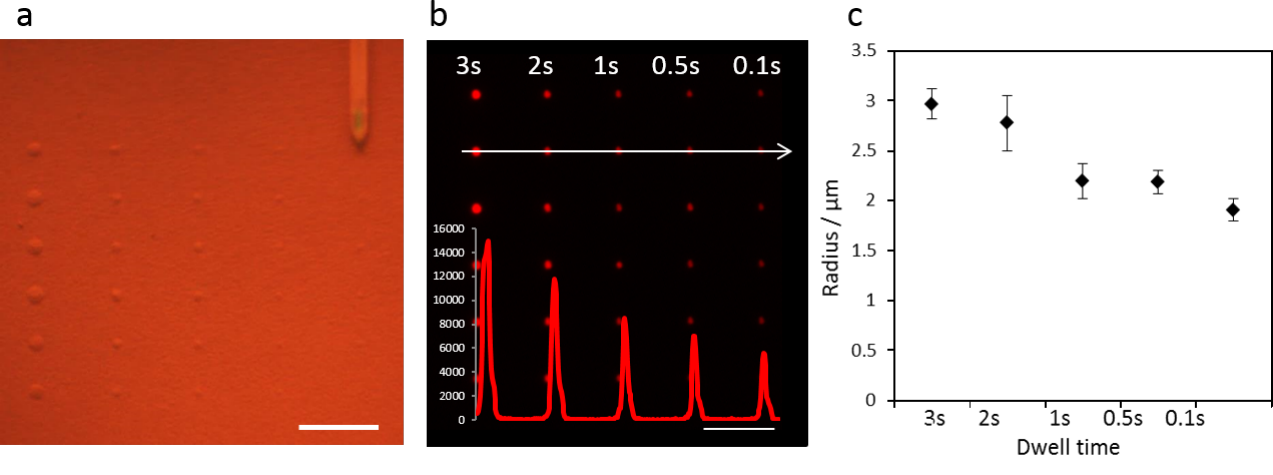
Phospholipid array on nanoporous HEMA-EDMA polymer. a) Phospholipid microcontact spotting on porous HEMA-EDMA with microchannel cantilevers imaged in situ on the lithography system. b) Fluorescence microscopy image of the DOPC phospholipid array doped with Rhodamine-PE (dwell times 3 to 0.1 s). The arrow indicates the intensity profile of dot features presented on the superimposed graph. Scale bar equals 50 µm. c) Relationship between spot radius and dwell time for the microarray shown in (b).

In order to establish best conditions for microchannel cantilever spotting (µCS) of phospholipids with the SPTs, we used simple phospholipid ink composed of DOPC [1,2-dioleoyl-*sn*-glycero-3-phosphocholine] doped with Rhodamine-PE [1,2-dioleoyl-*sn*-glycero-3-phosphoethanolamine-*N*-(lissamine rhodamine B sulfonyl) (ammonium salt)]. The ink was prepared by mixing DOPC and Rhodamine-PE to achieve 1 µM lipid content in chloroform with 1 mol % fluorescent dye admixture. The chloroform was evaporated and lipids were reconstituted with an ethanol and glycerol mixture in 7:3 v/v ratio. The ink was loaded onto a freshly oxygen plasma activated SPT and directly used for spotting. When varying the contact time between SPT and substrate (dwell time), different feature sizes can be obtained (Figure 1). The diameter of the spotted features varies from 6 +/− 0.3 µm to 4 +/− 0.2 µm for dwell times from 3 to 0.1 s. Interestingly, not only feature size, but also fluorescence intensity varies with dwell time (Figure 1b). This can be explained by the 3D nature of the features as they are actually embedded in the porous HEMA-EDMA substrate [10]. While on a flat glass substrate, features can only grow into 2D with dwell time, while in the present case features will also grow into the depth of the HEMA-EDMA film, resulting in brighter fluorescence as the ink below the feature surface also contributes to the signal.

### Protein and antibody binding onto lipid arrays

**Figure 2:**
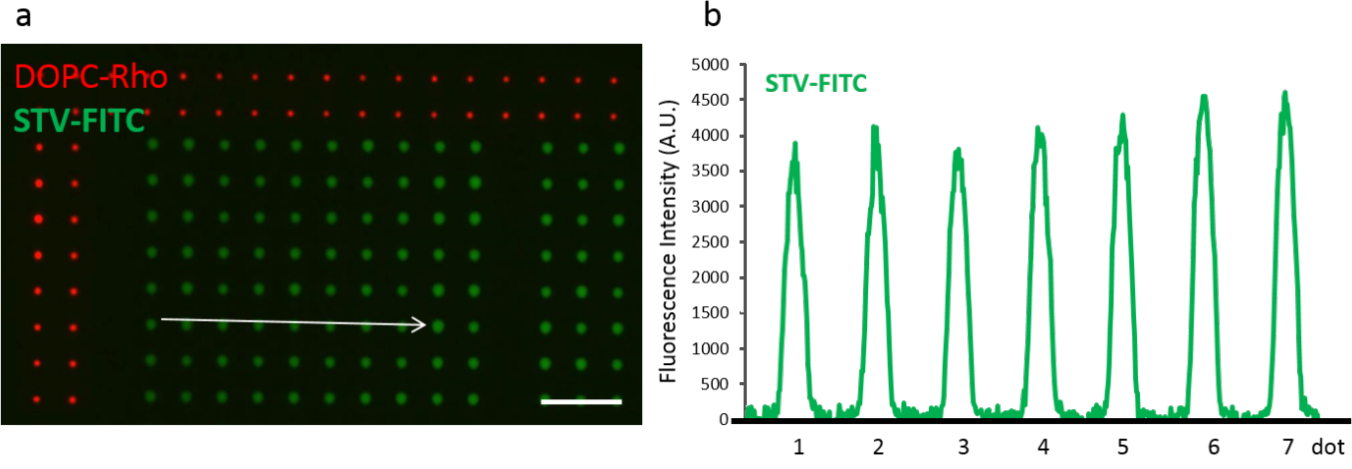
a) Fluorescence image of the STV-FITC binding Biotin-PE containing arrays on the nanoporous HEMA-EDMA substrate. DOPC doped with Rhodamine PE (red) was patterned around biotin array for optical reference with dwell time set to 1 s. Spotting dwell time for Biotin-PE containing arrays was set to 2 s. Scale bar equals 50 µm. b) The graph shows the fluorescence intensity after background subtraction of dots indicated by an arrow.

The selective binding of proteins to the lipid arrays on HEMA polymer was first tested using fluorophore labelled streptavidin (STV-FITC) for the coupling with biotin headgroups. Biotin lipid arrays were prepared by admixing 4 mol % biotin lipid (1,2-dipalmitoyl-*sn*-glycero-3-phosphoethanolamine-*N*-(cap biotinyl)) into the DOPC carrier. It was shown previously [24], that this percentage admixture of biotin saturates the number of available biotin headgroups for protein binding [24]. After spotting, the arrays were blocked with 10% bovine serum albumin (BSA) to reduce unspecific binding of the protein to the polymer. Next, samples were washed and incubated for 1 h with 0.5 µg solution of STV-FITC in phosphate buffered saline (PBS). The observed STV-FITC fluorescence is homogenous over the whole arrays, indicating an even distribution of biotin headgroups embedded in the nanoporous HEMA-EDMA (Figure 2).

**Figure 3:**
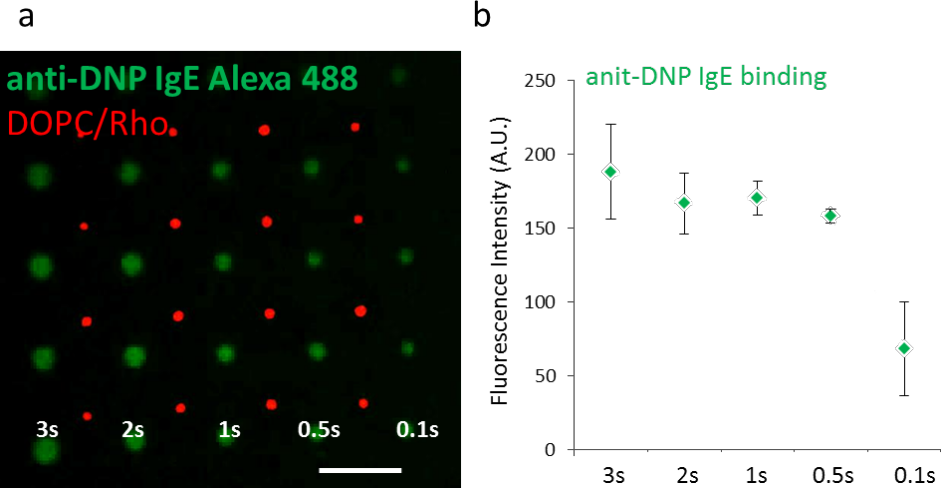
a) Fluorescence microscopy image of an anti-DNP IgE-Alexa 488 antibody (green) bound to an array of DNP-lipids on nanoporous HEMA-EDMA substrate. DOPC doped with Rhodamine-PE was patterned on the same sample as optical reference (red) with dwell time set to 1 s. Scale bar equals 50 µm. b) Fluorescence intensity of *n* = 5 dots features per dwell time of DNP-array (after background subtraction) presented in a) after binding the anti-DNP IgE-Alexa 488 antibody.

To assess the accessibility of lipid based allergen arrays on nanoporous HEM-EDMA A substrates to antibodies, we employed the established lipid/antibody pair DNP-cap-PE and anti-DNP IgE antibody. Previously, DNP-lipid recognition by a specific antibody was tested on glass supports [17], while on HEMA-EDMA polymer antibody binding was tested on covalently bound DNP-azide ink [10]. Here, allergen arrays were prepared by using 10 mol % admixture of DNP-cap-PE into DOPC lipid. As an optical reference DOPC doped with rhodamine was patterned in-between DNP-lipid structures (Figure 3a). After blocking with 10% BSA and subsequent washing, samples were incubated with anti-DNP IgE antibody labelled with Alexa 488 dye for direct visualization. Plotting of the mean fluorescence intensity against dwell time (Figure 3b) indicates a slight dependence of fluorescence intensity on dwell time (suggesting the infiltrated nature of the features as explained above). Though the difference between 2 s, 1 s and 0.5 s dwell time is not as strongly pronounced as in the case of the DOPC ink (Figure 1b), this dependency overall suggests that the antibody can access binding sites within the HEMA-EDMA substrate.

To test the binding of a more complex, functional protein on the HEMA-EDMA based lipid arrays, we chose AR^biot^. Binding of AR^biot^ to the HEMA-EDMA membrane was performed in the presence of dihydrotestosterone hormone (DHT). The hormone addition is needed to assure receptor stability as the AR is known to be very unstable as an aporeceptor [25]. Firstly, biotin carrying arrays for AR^biot^ binding were prepared as described in the previous section with an admixing of 4 mol % biotin lipid into DOPC carrier. Next, after blocking with 10% BSA and binding of fluorescently labeled STV-Cy3, arrays were washed with PBS and blocked again with 10% BSA to prepare membranes for AR^biot^ binding. The coating of arrays with labeled STV allowed for direct visualization of the biotin arrays. In the last washing step after blocking with BSA dihydrotestosterone (DHT, 10^−4^ M) was added to the buffer in order to prepare the array for the binding of AR^biot^ onto the STV-Cy3–biotin arrays [25]. Next, freshly purified AR^biot^ was loaded onto the sample surface. After overnight incubation at 4 °C, arrays were washed with PBS containing 10^−4^ DHT to maintain the AR stability. Binding of AR^biot^ was detected via indirect immunofluorescence. First samples were blocked with fetal calf serum (FCS) for 1 h at room temperature (RT), then washed with PBS containing 10^−4^ DHT and loaded for 1 h with anti-AR antibody.

**Figure 4:**
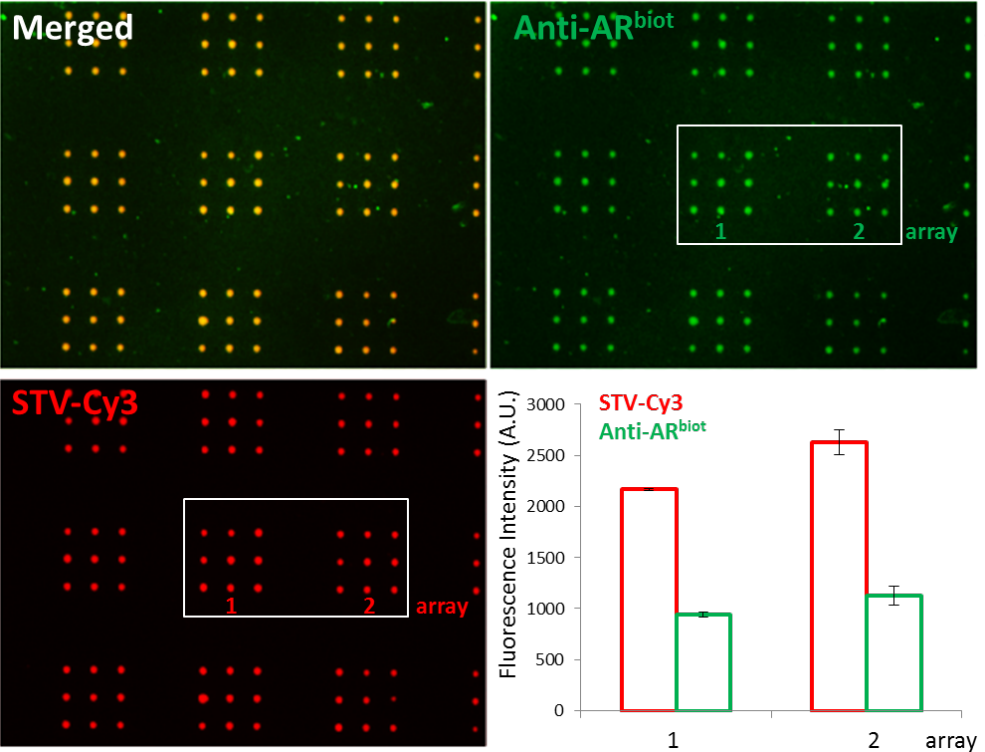
Fluorescence microscopy images of AR^biot^ binding onto STV-Cy3 coated biotin arrays on nanoporous HEMA-EDMA substrate. The STV-Cy3 was pre-bound onto biotin lipid spots (red), AR^biot^ was detected by immunostaining (green). Scale bars equal 50 µm. All arrays were spotted with a dwell time of 2 s. The graph presents intensity profiles of *n* = 9 dot features of two arrays, after STV-Cy3 (red points) and AR^biot^ binding (green points) (after background subtraction).

After another washing step, the sample was incubated with a secondary antibody labeled with FITC, for 15 min, washed with PBS containing 10^−4^ DHT and imaged with fluorescence microscope. Figure 4 shows biotin arrays after binding of STV-Cy3 (Texas Red channel) and AR^biot^, detected with indirect immunofluorescence (FITC channel). Binding of STV-Cy3 served as an optical reference to easily localize the lipid arrays on the sample. Fluorescence signal after antibody binding on AR^biot^ coated arrays is homogenous, indicating an even distribution of the receptor bound to STV-biotin arrays embedded in the HEMA-EDMA (Figure 4, graph).

## Conclusion

We present a novel platform for binding studies of functional molecules based on lipid arrays embedded in a nanoporous hydrophilic polymer substrate. The microcontact spotting approach used to pattern the functional lipid inks into arrays allows for the multiplexing of different inks into single arrays within the polymer mesh. Spotting with SPT probes on HEMA-EDMA polymer increases the spatial resolution of lipid pattern as compared to conventional spotting/ink jet printing on 3D substrates, such as PVDF or nitrocellulose membranes, from 50–300 µm in current commercial setups [26–27] to the presented average 6 µm features by µCS. The 3D nature of the spotted features (enabled by the infiltration of the ink into the nanoporous polymer) results in a higher signal intensity compared to similar arrays on strictly 2D substrates as glass [9–10]. As of now it is still unknown, just like in case of lipids spotted on PVDF membranes [7], how the polymer mesh accommodates lipid geometry and packaging, and how it affects the accessibility of the target lipid regions for the binding of proteins and antibodies. However, the infiltrated lipid arrays show high stability against washing steps, while still being accessible for protein and antibody binding. Even complex receptor protein structures of AR^biot^ remain intact upon binding to the lipid arrays and are still accessible for the indirect immunofluorescence detection. The described model system and its demonstrated feasibility exhibit a high potential for follow-up studies. Even though the model is still very simplified, its ability to reduce the complexity of interactions for the study of specific aspects of AR function in protein complexes has been demonstrated. This setup may allow for the investigation of differences between various protein complexes isolated from pathological samples from mice models and patient samples. Antibody based detection approaches could be suitable to analyze the levels of AR proteins bound onto the lipid arrays. The described model system would thereby have a significant impact on the therapeutic treatment for breast and prostate cancer patients.

Keeping in mind previous applications of HEMA polymer in creating superhydrophilic–superhydrophobic micropatterned surfaces for cell patterning [28] and cell-screening applications [29–30], the addition of lipid arraying to the portfolio of HEMA polymer applications emphasizes the use of this solid substrate as a very attractive platform for a variety of biomedical applications. The combination of microfluidic settings and phospholipid arrays on solid supports like HEMA polymer may also prove fruitful for a multitude of sensing applications.

## Experimental

### Substrate preparation

Fabrication of alkyne HEMA-EDMA polymer ﬁlm was described in our previous work [9–10]. Brieﬂy, a 12.5 µm thin, hydrophilic porous polymer ﬁlm was ﬁrstly prepared on a glass substrate by using photoinitiated copolymerization of 2-hydroxyethyl methacrylate and ethylene dimethacrylate in the presence of porogens (1-decanol and cyclohexanol) [29]. This procedure leads to the formation of a thin ﬁlm of a highly cross-linked and porous (due to the presence of porogens) hydrophilic polymethacrylate layer. The porous structure is a permeable network of interconnected polymer nanoglobules with pores of around 150 nm.

### Microcontact spotting

The patterning of lipid arrays was performed on a NLP 2000 system (NanoInk, USA) with oxygen plasma activated (0.2 mbar O_2_, 2 min) SPT probes (SPT-S-C10S, BioForce Nanosciences, USA). All lipids were purchased from Avanti Polar Lipids. Ink mixtures were prepared by admixing various lipids to DOPC carrier lipid: DOPC (20 mg/mL) and Rhodamine-PE (1 mg/mL) or Biotin-PE (1 mg/mL) or DNP-cap-PE (10 mg/mL) were used to prepare a 1 µM total lipid content ink mixture, sonicated together, air dried to remove the chloroform solvent and reconstituted with ethanol and glycerol (87%) in a 7:3 v/v ratio. Glycerol was added to prevent the premature drying of the ink on the SPT. Probes were filled with 0.5 µL ink, mounted onto the probe holder and used directly for spotting on unmodified poly(2-hydroxyethyl methacrylate-*co*-ethylene dimethacrylate) support (HEMA). Spotting was performed at 55–65% relative humidity. All patterning procedures were carried out with the sample stage tilted by 8° with respect to the SPT tip to minimize the probability of a contact between the ink reservoir and the sample surface. For all patterns, except the ones used for spot size versus dwell time analysis, a dwell time of 2 s was used for target patterns and 1 s for optical reference patterns.

### Protein binding

Streptavidin-FITC (Streptavidin, Fluorescein Conjugate) was purchased from Calbiochem and Streptavidin-Cy3 (recombinant from *Streptomyces avidinii*, Cy3 labelled) was purchased from Sigma. Prior to protein binding on biotin lipid arrays, samples were blocked in 10% BSA (Sigma-Aldrich) solution in PBS (Gibco) for 20 min at room temperature. After washing with PBS, 0.5 µg solution of STV-Cy3 in PBS was incubated for 30 min on the substrate. Samples were typically washed with 100 µL PBS 3× and imaged under fluorescence microscope (Nikon Instruments Inc.).

AR^biot^ purification and binding was done as follows: Sf9 [23] cells were cultured with continuous shaking at 110 rpm at a density of 10^9^ and a temperature of 27 °C in SF900 II medium (Invitrogen) supplemented with 5% fetal calf serum (FCS). One day prior to infection the medium was changed to serum-free SF900 II medium. Cells were infected with baculovirus stocks typically at MOIs of 5–7. 72 h post-infection cells were treated with DHT (10^−4^ M) for 3 h. The infected SF9 cells were lysed on ice in lysis buffer (20 mM HEPES pH 7.4, 400 mM NaCl, 1 mM β-mercaptoethanol, 1 mM ZnCl_2_, 1 mM PMSF, 1 mM Na_2_V0_3_, 10 μL protease inhibitor cocktail (Roche Mannheim, Germany) per mL) with 10^−4^ M DHT and an equivalent amount of ethanol in a control purification. The lysates were then centrifuged for 10 min at 14,000*g* at 4 °C. Isolation of the biotin-tagged AR samples was performed on a streptavidin–mutein-matrix (Roche Mannheim, Germany) according to the manufacturer's instructions at 4 °C [18]. Freshly purified protein was quantified for total protein content. The concentration of the protein was measured at 280 nm by using the specific program 1 A/cm = 1 mg from the spectrophotometer Nanodrop Lite (Thermo Scientific) in duplicate and with a solution of elution buffer (10^−4^ DHT) as the blank. After blocking the sample surface with 10% BSA solution in PBS for 30 min at RT, samples were incubated with 0.5 µg solution of STV or STV-Cy3 in PBS for 30 min at room temperature. Next, samples were washed 2× with 100 µL PBS, and a final washing step was carried out with PBS containing 10^−4^ DHT. Then, 200 µL of freshly purified AR^biot^ (0.45 µg/µL) was loaded onto the sample. Samples were incubated over night at 4 °C for optimal protein binding. Samples were then washed 3 times with 100 µL PBS containing 10^−4^ M DHT and blocked again with 100 µL fetal bovine serum for 1 h at RT. Thereafter, samples were washed again 3 times with 100 µL PBS containing 10^−4^ M DHT and incubated with 100 µL primary anti-AR antibody (AR F39.4.1 from Santa Cruz Biotechnology, INC., sc-52309, 1:200 in PBS containing 10^−4^ M DHT) for 1–2 h at room temperature. Next, the samples were washed 3 times with 100 μL PBS containing 10^−4^ M DHT and incubated for 15 min. with 100 μL of the secondary antibody (Goat anti-Mouse IgG (H+L) Secondary Antibody, Alexa Fluor 488, ThermoFisher, A-11001, diluted in a ratio of 0.5:400 onto PBS containing 10^−4^ M DHT). Finally, the membranes were washed 3 times with PBS containig 10^−4^ DHT and imaged under upright fluorescence microscope (Nikon Instruments Inc.).

### Antibody binding

DNP-lipid arrays were blocked with 10% BSA in PBS for 30 min at room temperature. After washing 3 times with PBS, 200 µL of 5 µg/mL of anti-DNP IgE-Alexa 488 antibody in PBS was applied. After 1 h incubation, samples were washed 3 times in PBS and imaged under fluorescence microscope (Nikon Instruments Inc.).

### Fluorescence microscopy and quantification of signal

Fluorescence microscopy was carried out on an Eclipse 80i upright microscope (Nikon Instruments Inc.). Fluorescence images were taken with 10× or 20× objectives. Images were recorded with a CoolSNAP HQ2 camera (Photometrics). Patterns were aligned for imaging by using alignment markers scratched onto the polymer surface. The bleaching of fluorophores was minimized (especially for quantitative measurements) by first focusing on the alignment mark before exposing the patterned area to light, and the lamp intensity was kept at a minimum. Fluorescence intensity profiles were generated by using NIS-AR software installed on the microscope PC (Nikon Instruments Inc.). For measurements the patterned areas of interest were marked, and fluorescence intensity was automatically recorded. Mean fluorescence intensity after background extraction was calculated using the onboard NIS-AR software as previously described in [17].
